# Bibliometric analysis of the top 100 cited articles in head and neck radiology

**DOI:** 10.1177/20584601211001815

**Published:** 2021-03-12

**Authors:** Aye MM Oo, Timothy SM ChuT

**Affiliations:** 1 South Tyneside District Hospital, South Shields, UK; 2 School of Medical Education, Newcastle University, Newcastle Upon Tyne, UK

**Keywords:** Radiology, head and neck cancer, bibliometrics, citation analysis, diagnostic imaging, head and neck surgery

## Abstract

**Background:**

Bibliometric analysis is commonly used to identify influential research within a given topic.

**Purpose:**

To identify the 100 top-cited articles in head and neck radiology, analyse the history and trends in head and neck imaging research, and understand what constitutes a highly cited work.

**Material and Methods:**

A literature search was performed on the Thomson Reuters Web of Science using pre-defined search terms. The results were ranked according to citation count and screened to create a single database. The information included in the database were: Web of Science citations, year published, first author, primary institution, country of origin, journal, journal impact factor, title, study design, study focus and modality.

**Results:**

24,664 eligible papers were returned. Citations for the 100 top-cited articles ranged from 115 to 1185, and citations per year ranged from 3.5 to 197.5. More than half of the articles were published in the 2000s (*n* = 67). Radiology has the greatest number of publications (*n* = 22), followed by Journal of Nuclear Medicine (*n* = 14). Positron Emission Tomography (*n* = 56) was the most commonly studied modality, followed by Magnetic Resonance (*n* = 40) and Computed Tomography (*n* = 31). The most common topics of publication were diagnosis (*n* = 63), followed by prognosis (*n* = 16).

**Conclusion:**

This study provides insights into the most influential research in head and neck radiology in the current time. It also serves as a guide to the characteristics of a highly cited work in this field.

## Introduction

Bibliometrics is the statistical evaluation of publications to allow for the assessment of impact, research performance and author productivity. Citation count is one of the bibliometric parameters which allows us to identify the most influential articles and their impacts on their fields.^1–3^ Bibliometric analysis is useful in identifying trends in a specific field of research and directions for future research.

There have been many studies investigating the most frequently cited articles in different medical fields.^4–7^ Within the field of radiology, there have been articles evaluating the top cited papers for a specific journal,^8,9^ for the field of radiology^10–12^ and for radiology subspecialties.^13–15^

To the best of our knowledge, there have been no bibliometric analyses focusing on the area of head and neck radiology that includes all types of studies. Therefore, the aim of this study was to identify and examine the top 100 most cited articles on head and neck radiology across all scientific journals. By not restricting our search to the articles published in radiology-specific journals as performed by other studies,^11,12^ we complied a comprehensive list of most influential articles in head and neck radiology. This study presents the most influential publications to date in the field to provide a perspective on the current research trends and future research directions.

## Material and Methods

A bibliometric analysis of the most highly cited articles in Head and Neck Radiology was conducted in April 2020. We performed a search on Thomson Reuters Web of Science (WOS) using the following key terms:

· Head and neck AND radiology OR

· Head and neck AND imaging OR

· Head and neck AND (XR OR X ray OR radiograph) OR

· Head and neck AND (CT OR computed tomography) OR

· Head and neck AND (MR OR magnetic resonance) OR

· Head and neck AND (US OR ultrasound OR sonography) OR

· Head and neck AND (IR OR interventional radiology) OR

· Head and neck AND nuclear imaging OR

· Head and neck AND nuclear medicine

This returned a total of 24,664 articles and all articles were included, regardless of language or electronic availability of articles. The results were then sorted by the number of citations from the most cited to the least cited. This method was developed by Paladugu et al.^16^ and was used in several other studies.^13,17,18^ Each article was assessed for inclusion. Articles were included if they focused on diagnostic imaging interpretation, imaging technique, comparison of modalities, utility and role of different imaging modalities or trends in head and neck radiology. Articles that focused on head and neck pathologies other than head and neck cancer such as thyroid disorders were also included. Articles were excluded if they did not pertain to head and neck radiology, if the primary focus was not on head and neck, or if they focused on neuroimaging or dental imaging. Articles that mainly focused on therapeutic radiology were also excluded.

The top 100 most cited articles were identified and compiled into a single database. The database included: WOS citations, year published, first author, primary institution, country of origin, journal, journal impact factor, title, study design, study focus and modality.

## Results

The top 100 most cited articles, with a mean citation number of 181.73 and median of 156, are shown in Table 1. The number of citations ranged from 115 to 1185.

**Table 1.** The 100 top-cited articles in head and neck radiology ranked in descending number of citations.

Rank	Citations	Average citations per year	Average citations per year in the first 5 years since publication	First author	Year	Title
1	1185	197.5	221.8	Aerts HJWL	2014	Decoding tumour phenotype by noninvasive imaging using a quantitative radiomics approach
2	448	28.0	24.4	Daisne JF	2004	Tumor volume in pharyngolaryngeal squamous cell carcinoma: comparison at CT, MR imaging, and FDG PET and validation with surgical specimen

3	352	18.5	6.8	Wang JC	2001	Head and neck lesions: characterization with diffusion-weighted echo-planar MR imaging
4	317	21.1	22.0	Eschmann SM	2005	Prognostic impact of hypoxia imaging with F-18-misonidazole PET in non-small cell lung cancer and head and neck cancer before radiotherapy

5	315	11.3	10	Som PM	1992	Detection of metastasis in cervical lymph-nodes – CT and MR criteria and differential diagnosis
6	306	13.9	13.2	Adams S	1998	Prospective comparison of F-18-FDG PET with conventional imaging modalities (CT, MRI, US) in lymph node staging of head and neck cancer

7	287	17.9	32	Schoder H	2004	Head and neck cancer: clinical usefulness and accuracy of PET/CT image fusion
8	260	18.6	19	Rajendran JG	2006	Tumor hypoxia imaging with [F-18] fluoromisonidazole positron emission tomography in head and neck cancer
9	238	7.4	6.8	Minn H	1988	Fluorodeoxyglucose imaging: a method to assess the proliferative activity of human cancer in vivo. Comparison with DNA flow cytometry in head and neck tumors.

10	229	10.4	9.4	Curtin HD	1998	Comparison of CT and MR imaging in staging of neck metastases
11	228	20.7	21	Kim S	2009	Diffusion-weighted magnetic resonance imaging for predicting and detecting early response to chemoradiation therapy of squamous cell carcinomas of the head and neck

12	223	15.9	17.2	Razek AAKA	2006	Role of diffusion-weighted MR imaging in cervical lymphadenopathy
13	218	14.5	22.2	Branstetter BF	2005	Head and neck malignancy: is PET/CT more accurate than PET or CT alone?
13	218	12.8	7.2	Sumi M	2003	Discrimination of metastatic cervical lymph nodes with diffusion-weighted MR imaging in patients with head and neck cancer

15	216	10.3	9.4	Hustinx R	1999	Dual time point fluorine-18 fluorodeoxyglucose positron emission tomography: a potential method to differentiate malignancy from inflammation and normal tissue in the head and neck
16	215	8.0	16.6	Jabour BA	1993	Extracranial head and neck: PET imaging with 2-[F-18]fluoro-2-deoxy-D-glucose and MR imaging correlation
17	211	5.7	5.4	Mancuso AA	1983	Computed tomography of cervical and retropharyngeal lymph nodes: normal anatomy, variants of normal, and applications in staging head and neck cancer. Part II: pathology.

18	208	18.9	21.4	Vandecaveye V	2009	Head and neck squamous cell carcinoma: value of diffusion-weighted MR imaging for nodal staging

19	201	11.2	13.6	Brun E	2002	FDG PET studies during treatment: prediction of therapy outcome in head and neck squamous cell carcinoma
19	201	7.4	7.4	Vandenbrekel MWM	1993	Modern imaging techniques and ultrasound-guided aspiration cytology for the assessment of neck node metastases: a prospective comparative study

21	200	22.2	19	Gupta T	2011	Diagnostic performance of post-treatment FDG PET or FDG PET/CT imaging in head and neck cancer: a systematic review and meta-analysis

21	200	11.8	7.4	Chan BK	2003	Common and uncommon sonographic features of papillary thyroid carcinoma
23	198	7.9	12.6	Laubenbacher C	1995	Comparison of fluorine-18-fluorodeoxyglucose PET, MRI and endoscopy for staging head and neck squamous-cell carcinomas.

24	196	16.3	15	Kyzas PA	2008	F-18-fluorodeoxyglucose positron emission tomography to evaluate cervical node metastases in patients with head and neck squamous cell carcinoma: a meta-analysis


25	195	10.3	11	Greven KM	2001	Serial positron emission tomography scans following radiation therapy of patients with head and neck cancer
26	194	12.9	11.4	Ng SH	2005	F-18-FDG PET and CT/MRI in oral cavity squamous cell carcinoma: a prospective study of 124 patients with histologic correlation

27	193	9.7	4.6	Som PM	2000	Imaging-based nodal classification for evaluation of neck metastatic adenopathy
28	189	14.5	11.2	de Bondt RBJ	2007	Detection of lymph node metastases in head and neck cancer: a meta-analysis comparing US, USgFNAC, CT and MR imaging
29	188	14.5	19.4	Beer AJ	2007	[F-18]Galacto-RGD positron emission tomography for imaging of alpha v beta 3 expression on the neovasculature in patients with squamous cell carcinoma of the head and neck

29	188	6.5	9.0	Okada J	1991	The use of FDG-PET in the detection and management of malignant lymphoma: correlation of uptake with prognosis
31	187	7.8	13.4	Anzai Y	1996	Recurrence of head and neck cancer after surgery or irradiation: prospective comparison of 2-deoxy-2-[F-18]fluoro-D-glucose PET and MR imaging diagnoses

32	186	7.4	15	Braams JW	1995	Detection of lymph node metastases of squamous-cell cancer of the head and neck with FDG-PET and MRI
33	184	7.1	11.4	Anzai Y	1994	Initial clinical experience with dextran-coated superparamagnetic iron oxide for detection of lymph node metastases in patients with head and neck cancer

34	182	45.5	49	Mehanna H	2016	PET-CT surveillance versus neck dissection in advanced head and neck cancer

34	182	15.2	18	Ong SC	2008	Clinical utility of F-18-FDG PET/CT in assessing the neck after concurrent chemoradiotherapy for locoregional advanced head and neck cancer

36	179	6.4	6.8	Yousem DM	1992	Central nodal necrosis and extracapsular neoplastic spread in cervical lymph nodes: MR imaging versus CT.
37	178	22.3	26.2	Mortensen LS	2012	FAZA PET/CT hypoxia imaging in patients with squamous cell carcinoma of the head and neck treated with radiotherapy: results from the DAHANCA 24 trial

38	177	6.3	9.8	Bailet JW	1992	Positron emission tomography: a new, precise imaging modality for detection of primary head and neck tumors and assessment of cervical adenopathy
39	175	6.7	12.4	Rege S	1994	Use of positron emission tomography with fluorodeoxyglucose in patients with extracranial head and neck cancers
40	174	10.9	7.6	Rusthoven KE	2004	The role of fluorodeoxyglucose positron emission tomography in cervical lymph node metastases from an unknown primary tumor

41	171	5.9	4.4	Vandenbrekel MWM	1991	Occult metastatic neck disease: detection with US and US-guided fine-needle aspiration cytology

42	169	13	15.8	Grosu AL	2007	Hypoxia imaging with FAZA-PET and theoretical considerations with regard to dose painting for individualization of radiotherapy in patients with head and neck cancer

42	169	11.3	8.8	Rosario PWS	2005	Ultrasonographic differentiation between metastatic and benign lymph nodes in patients with papillary thyroid carcinoma

42	169	6.5	10.8	Greven KM	1994	Positron emission tomography of patients with head and neck carcinoma before and after high dose irradiation
45	167	20.9	22.6	Zips D	2012	Exploratory prospective trial of hypoxia-specific PET imaging during radiochemotherapy in patients with locally advanced head-and-neck cancer
46	162	11.6	11.6	Ng SH	2006	Prospective study of [F-18] fluorodeoxyglucose positron emission tomography and computed tomography and magnetic resonance imaging in oral cavity squamous cell carcinoma with palpably negative neck

47	161	10.7	11.6	Nakamoto Y	2005	Normal FDG distribution patterns in the head and neck: PET/CT evaluation
48	160	12.3	12.2	Vandecaveye V	2007	Detection of head and neck squamous cell carcinoma with diffusion weighted MRI after (chemo)radiotherapy: correlation between radiologic and histopathologic findings

49	159	12.2	10.2	Johnson NA	2007	Parathyroid imaging: technique and role in the preoperative evaluation of primary hyperparathyroidism
50	156	11.1	12	Schoder H	2006	F-18-FDG PET/CT for detecting nodal metastases in patients with oral cancer staged N0 by clinical examination and CT/MRI
50	156	7.8	12.2	Lowe VJ	2000	Surveillance for recurrent head and neck cancer using positron emission tomography
52	155	12.9	17.4	Nehmeh SA	2008	Reproducibility of intratumor distribution of F-18-fluoromisonidazole in head and neck cancer
53	154	9.6	8.2	King AD	2004	Necrosis in metastatic neck nodes: diagnostic accuracy of CT, MR imaging, and US
54	153	10.9	11.4	Daly MJ	2006	Intraoperative cone-beam CT for guidance of head and neck surgery: assessment of dose and image quality using a C-arm prototype

55	151	18.9	20	Thoeny HC	2012	Diffusion-weighted MR imaging in the head and neck
55	151	13.7	13	Dirix P	2009	Dose painting in radiotherapy for head and neck squamous cell carcinoma: value of repeated functional imaging with F-18-FDG PET, F-18-Fluoromisonidazole PET, diffusion-weighted MRI, and dynamic contrast-enhanced MRI

57	149	13.5	14.4	Abgral R	2009	Does F-18-FDG PET/CT improve the detection of posttreatment recurrence of head and neck squamous cell carcinoma in patients negative for disease on clinical follow-up?
58	148	13.5	13.4	Chung MK	2009	Metabolic tumor volume of [F-18]-fluorodeoxyglucose positron emission tomography/computed tomography predicts short-term outcome to radiotherapy with or without chemotherapy in pharyngeal cancer
59	147	12.3	7.6	Lee YYP	2008	Imaging of salivary gland tumours
59	147	10.5	9.8	Borjesson PKE	2006	Performance of immuno-positron emission tomography with zirconium-89-labeled chimeric monoclonal antibody U36 in the detection of lymph node metastases in head and neck cancer patients

59	147	5.9	9.2	Becker M	1995	Neoplastic invasion of the laryngeal cartilage: comparison of MR imaging and CT with histopathologic correlation.
62	145	18.1	20.6	Dibble EH	2012	F-18-FDG metabolic tumor volume and total glycolytic activity of oral cavity and oropharyngeal squamous cell cancer: adding value to clinical staging

62	145	13.2	15.4	La TH	2009	Metabolic tumor volume predicts for recurrence and death in head-and-neck cancer
62	145	5.8	5.2	Steinkamp HJ	1995	Cervical lymphadenopathy: ratio of long- to short-axis diameter as a predictor of malignancy.
65	144	9.6	14.2	Schwartz DL	2005	FDG-PET/CT imaging for preradiotherapy staging of head-and-neck squamous cell carcinoma
65	144	7.2	6.8	Stuckensen T	2000	Staging of the neck in patients with oral cavity squamous cell carcinomas: a prospective comparison of PET, ultrasound, CT and MRI

67	142	9.5	6	Ahuja AT	2005	Sonographic evaluation of cervical lymph nodes
68	139	13.9	14.6	Lonneux M	2010	Positron emission tomography with [F-18] fluorodeoxyglucose improves staging and patient management in patients with head and neck squamous cell carcinoma: a multicenter prospective study

69	137	9.1	9.2	Yao M	2005	The role of FDG PET in management of neck metastasis from head-and-neck cancer after definitive radiation treatment
69	137	8.1	5	Ahuja A	2003	Sonography of neck lymph nodes. Part II: abnormal lymph nodes
71	136	5.4	11.4	Lapela M	1995	Head and neck cancer: detection of recurrence with PET and 2-[F-18]fluoro-2-deoxy-D-glucose
72	135	12.3	15.6	Holzapfel K	2009	Value of diffusion-weighted MR imaging in the differentiation between benign and malignant cervical lymph nodes
73	134	10.3	8.4	Razek AAKA	2007	Role of diffusion-weighted echo-planar MR imaging in differentiation of residual or recurrent head and neck tumors and posttreatment changes
73	134	8.4	8	Schwartz DL	2004	FDG-PET prediction of head and neck squamous cell cancer outcomes
75	133	11.1	12.2	Komar G	2008	F-18-EF5: a new PET tracer for imaging hypoxia in head and neck cancer
75	133	10.2	10.6	King AD	2007	Malignant cervical lymphadenopathy: diagnostic accuracy of diffusion-weighted MR imaging
75	133	3.6	4.2	Mancuso AA	1983	Computed tomography of cervical and retropharyngeal lymph nodes: normal anatomy, variants of normal, and applications in staging head and neck cancer. Part I: normal anatomy.

78	131	10.1	13.4	Souvatzoglou M	2007	Tumour hypoxia imaging with [F-18]FAZA PET in head and neck cancer patients: a pilot study

78	131	6.0	6.2	van den Brekel MWM	1998	The size of lymph nodes in the neck on sonograms as a radiologic criterion for metastasis: how reliable is it?

80	129	11.7	12.8	Schoder H	2009	PET monitoring of therapy response in head and neck squamous cell carcinoma
81	128	4.7	5	Baker LL	1993	Hemangiomas and vascular malformations of the head and neck: MR characterization.
82	127	7.9	9	Lehtio K	2004	Imaging perfusion and hypoxia with pet to predict radiotherapy response in head-and-neck cancer
82	127	3.6	3.8	Stevens MH	1985	Computed tomography of cervical lymph nodes. Staging and management of head and neck cancer.
84	126	9.0	11.4	Andrade RS	2006	Posttreatment assessment of response using FDG-PET/CT for patients treated with definitive radiation therapy for head and neck cancers
84	126	4.3	11.8	Barakos JA	1991	Orbit, skull base, and pharynx: contrast-enhanced fat suppression MR imaging
86	125	12.5	13.6	Kim S	2010	Prediction of response to chemoradiation therapy in squamous cell carcinomas of the head and neck using dynamic contrast-enhanced MR imaging

86	125	6.9	9.4	Stoeckli SJ	2002	Is there a role for positron emission tomography with 18F-fluorodeoxyglucose in the initial staging of nodal negative oral and oropharyngeal squamous cell carcinoma

86	125	5.4	6.6	Becker M	1997	Necrotizing fasciitis of the head and neck: role of CT in diagnosis and management
89	124	7.3	5	Hermans R	2003	Tumor perfusion rate determined noninvasively by dynamic computed tomography predicts outcome in head-and-neck cancer after radiotherapy
89	124	4.6	10.8	Haberkorn U	1993	Fluorodeoxyglucose imaging of advanced head and neck cancer after chemotherapy.
91	122	11.1	15.6	Holzapfel K	2009	Value of diffusion-weighted MR imaging in the differentiation between benign and malignant cervical lymph nodes
91	122	10.2	10.4	Srinivasan A	2008	Differentiation of benign and malignant pathology in the head and neck using 3T apparent diffusion coefficient values: early experience

93	121	8.1	7.8	Maeda M	2005	Usefulness of the apparent diffusion coefficient in line scan diffusion-weighted imaging for distinguishing between squamous cell carcinomas and malignant lymphomas of the head and neck

94	120	5.2	7.6	Pameijer FA	1997	Can pretreatment computed tomography predict local control in T3 squamous cell carcinoma of the glottic larynx treated with definitive radiotherapy?

95	118	14.8	17.4	Lim R	2012	F-18-FDG PET/CT metabolic tumor volume and total lesion glycolysis predict outcome in oropharyngeal squamous cell carcinoma
95	118	6.6	10.6	Mack MG	2002	Superparamagnetic iron oxide – enhanced MR imaging of head and neck lymph nodes
97	117	10.6	8.6	Yu H	2009	Coregistered FDG PET/CT-based textural characterization of head and neck cancer for radiation treatment planning

97	117	7.8	10.4	Hicks RJ	2005	Utility of FMISO PET in advanced head and neck cancer treated with chemoradiation incorporating a hypoxia-targeting chemotherapy agent
97	117	3.5	4	Lufkin R	1987	New needle for MR-guided aspiration cytology of the head and neck
100	115	11.5	13	Vandecaveye V	2010	Predictive value of diffusion-weighted magnetic resonance imaging during chemoradiotherapy for head and neck squamous cell carcinoma


### Citations per year

Citations per year ranged from 3.5 to 197.5 with a mean of 13.2 and a median of 10.6 per year. The article by Aerts HJWL et al.^19^ is the most cited article in the list and has the highest number of citations per year.

### Year of publication

**Fig. 1.**
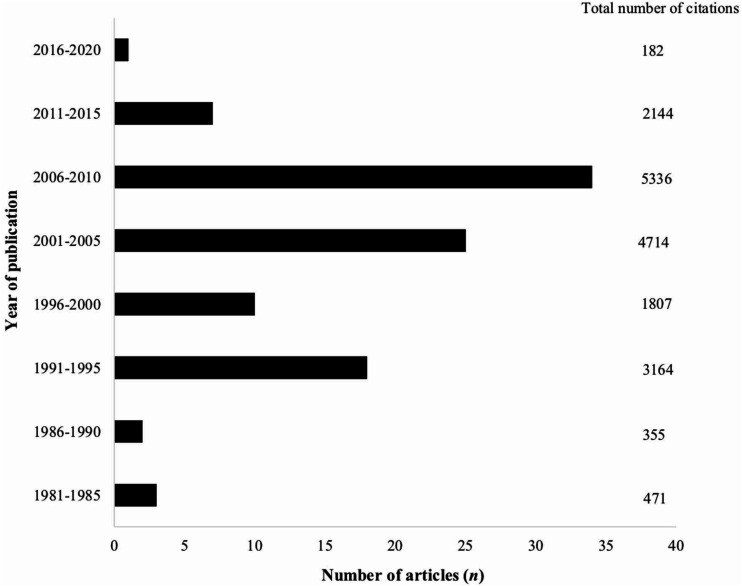
Distribution of the 100 top-cited articles in head and neck radiology and total number of citations by five-year span of publication.

The articles were published between 1983 and 2016; 2005 and 2009 had the greatest number of publications, with 10 articles each year. More than half of the articles on the list were published in the 2000s. Fig. 1 shows the distribution of the articles and the total number of citations by a five-year span of publication.

### Most common first author

There was a total of 83 first authors on the top 100 list. Amongst them, Vandecaveye V, Vandenbrekel MWM and Schoder H have the greatest number of articles, with three articles each. There were 14 authors that were first authors of more than one article to the top 100 list.

### Journals

The top 100 articles were published across 26 journals (Table 2). Radiology has the greatest number of publications (*n* = 22), followed by Journal of Nuclear Medicine (*n* = 14). New England Journal of Medicine had the highest impact factor of 70.67 and contributed to one article on the list.

**Table 2.** Journals in which the 100 top-cited articles in head and neck radiology were published.

Journal	Number of articles	Impact factor (2019)	Total number of citations
Radiology	22	7.931	4273
Journal of Nuclear Medicine	14	7.887	2370
International Journal of Radiation Oncology Biology Physics	10	5.859	1407
American Journal of Neuroradiology	7	3.381	979
American Journal of Roentgenology	5	3.013	926
Clinical Cancer Research	5	10.107	971
Cancer	4	5.742	756
Journal of Clinical Oncology	4	32.956	579
European Journal of Nuclear Medicine and Molecular Imaging	3	7.081	448
European Journal of Radiology	3	2.687	471
Head and Neck - Journal for the Sciences and Specialties of the head and neck	3	2.538	521
JAMA Otolaryngology-Head & Neck Surgery^a^	2	3.848	261
European Journal of Nuclear Medicine	2	-	522
European Radiology	2	4.101	338
Journal of Ultrasound in Medicine	2	1.759	369
Radiotherapy and Oncology	2	4.856	345
British Journal of Radiology	1	2.196	145
Clinical Radiology	1	2.118	137
European Archives of Oto-Rhino-Laryngology	1	1.809	201
IEEE Transactions on Medical Imaging	1	6.685	117
Journal of Cranio-Maxillofacial Surgery	1	1.766	144
Journal of the National Cancer Institute	1	11.577	196
Laryngoscope	1	2.465	177
Medical Physics	1	3.317	153
Nature Communications	1	12.121	1185
New England Journal Of Medicine	1	74.699	182

^a^Title change in 2013, previously “Archives of Otolaryngology-Head and Neck Surgery”.

### Country and institution of origin

**Fig. 2.**
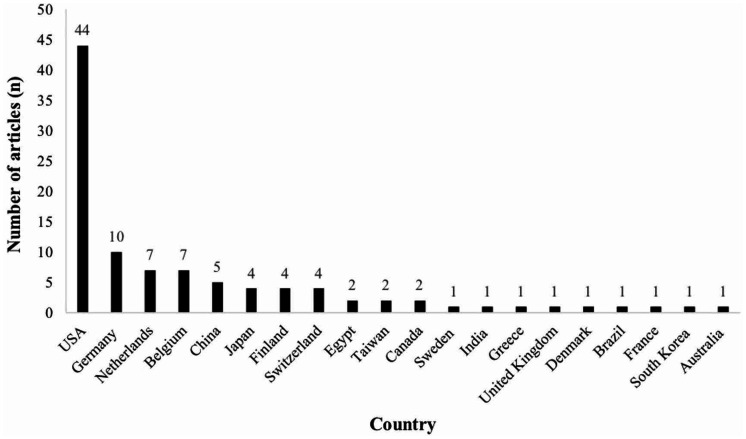
Country of the first author for 100 top-cited articles in head and neck radiology.

The United States (USA) has the greatest number of publications on the list (*n* = 44), followed by Germany (*n* = 10). The results are shown in Fig. 2. In terms of the affiliated academic institutions of the first authors on the list, Memorial Sloan Kettering Cancer Centre has the highest number of articles (*n* = 6), followed by University Hospital Leuven (*n* = 5) and University of California Los Angeles (*n* = 5).

### Study design

Study design was mostly prospective (*n* = 80), followed by reviews (*n* = 11), retrospective (*n* = 8) and a mix of prospective and retrospective (*n* = 1) (Table 3).

**Table 3.** Descriptors of the 100 top-cited articles in head and neck radiology.

	Descriptor	Frequency (*n*)
Study design	Prospective	80
	Review	11
	Retrospective	8
	Mixed	1
Modality	PET	56
24 articles focused on more than one modality	MR	40
	CT	31
	US	14
		
Primary topic/focus	Diagnosis	63
	Prognosis	16
	Treatment response	10
	Management	5
	Others	6
Related to head and neck cancer	Yes	92
	No	8

PET: Positron Emission Tomography; MR: Magnetic Resonance; CT: Computed Tomography; US: ultrasound.

### Topic

Imaging modality, primary topic/focus of the article and if the article was head and neck cancer related were also evaluated for all of the articles on the list. This is shown in Table 3.

## Discussion

The current bibliometric analysis offers some interesting insights into the field of head and neck radiology research, its history and how head and neck imaging has evolved over the years.

From our analysis, the most cited article was published in Nature Communications by Aerts HJWL et al. titled ‘Decoding tumour phenotype by non-invasive imaging using a quantitative radiomics approach’ in 2014.^19^ This article has also received the greatest number of citations per year, with an average of 197.5 citations per year. We noted that 90 out of 100 articles were published before 2010. This might bias the list in favour of the articles that had been published for a longer period of time and hence accumulated a larger number of citations. We have, therefore, included the average citations per year in our analysis (Table 1) for the articles on the top 100 list. Looking at the top 10 articles by average citations per year, six of the articles were published after 2010 while all of them were published after 2000.

Analysing the top three articles with the most citations per year, it was interesting to note that they were all published in the 2000s. Computed Tomography (CT) imaging was a recurring theme in all three articles and all of them focused on the clinical utility of non-invasive imaging in the clinical management of patients with head and neck cancer. These results may have reflected the trend in clinical medicine of this era, where imaging was being integrated into diagnosis and management alongside clinical examination.^20^

It was also noteworthy that the most cited article on the top 100 list (1185 citations) is approximately 6.5 times that of the mean (182) and 7.6 times that of the median (156). There is also a significant discrepancy between the most cited paper and the rest of the papers on the list (range, 115–448). We propose that this could be due to the increasing emphasis placed on evaluating the prognosis of the head and neck cancer patients using non-invasive imaging. Prognostic imaging was also discussed in 16 other articles on the top 100 list.

The top three journals (Radiology, Journal of Nuclear Medicine and International Journal of Radiation Oncology Biology Physics) with the most publications on the top 100 list are all journals in the field of radiology. This is in contrast to the findings of a previous bibliometric analysis focusing on head and neck cancer in general,^18^ which showed that the top contributing journals were high impact factor general medical journals such as the New England Journal of Medicine and Lancet. This difference might be due to the fact that we are focusing specifically on radiology in the head and neck region.

The journal impact factor is the average number of citations received by articles published in the journal in the last two years. By reviewing the impact factors of the journals on the top 100 list, we can evaluate the quality and influence of the top 100 cited articles. The higher the impact factor, the more influential the journal is in their field. This is supported by the fact that the three journals above are amongst the top 10 journals in the field of radiology by impact factor.^21^

Forty-four percent of the top 100 articles were from academic institutions in the USA. This was also seen in other bibliometric analyses in head and neck surgery^6,22^ and radiology.^12,13^ This might be attributed by the tendency of the USA authors to cite local papers and their influential research culture in medical training.^23,24^

From our analysis, 92 out of 100 articles were related to head and neck cancer. Eight other articles focused on other head and neck pathologies such as hyperparathyroidism, thyroid disorders, vascular malformations and hemangiomas. The prevalence of head and neck cancer-related articles in the top 100 list might be explained by the fact that cancer imaging is an important strand in head and neck radiology. Positron Emission Tomography (PET) seemed to be a recurring theme in all decades and was the most studied imaging modality. The popularity of these themes might be explained by the evolution of PET in oncologic imaging over the years.^25^ Examining the articles on the list by decade shows some trends in the progression of head and neck radiology research. Papers from the 1980s were mainly on diagnostic imaging with one article in the late 1980s discussing about fluorodeoxyglucose-PET.^26^ This decade also marked the beginning of extensive research on the use of PET in head and neck conditions, which continued into the next few decades. In the 1990s, studies compared different imaging modalities and compared these modalities with established screening and diagnosing methods. They were also evaluated for their clinical utility. This then gave way to studies that focused more on the clinical utility of the different imaging modalities, particularly PET. Several number of articles from this decade also looked into hypoxia imaging with PET.^27–30^ Finally, a trend towards using imaging for surveillance and treatment response was noted for studies published in the 2010s. It is expected that imaging modalities relating to early detection and surveillance of head and neck cancer will continue to be a main focus of research in the field of head and neck radiology in the future. This is because many of these imaging modalities now play an integral role in the diagnostic and surveillance pathways of head and neck cancer. With the increasing interests on non-invasive investigative techniques, it is predicted that imaging modalities such as PET and MRI will continue to be extensively researched.

This is the first bibliometric analysis focusing on head and neck radiology. Having such a list can provide radiologists and researchers with information on the most influential papers in the field of head and neck radiology. It also provides insights into how head and neck radiology has evolved over the decades and the advances in head and neck radiology research.

Our study has limitations as with all bibliometric analyses. First, our main limitation is the search terms that were used in this study. Articles that did not use ‘head and neck’ as a keyword may have been excluded from our database. Similarly, our database was restricted to articles, which included ‘radiology’ or ‘imaging’ or one of the imaging modalities mentioned above. Second, citation numbers often differ between databases such as WOS, Scopus, PubMed and Google Scholar. We chose WOS as it is the most commonly used database for bibliometric analyses. Finally, with our study being a bibliometric analysis, there is a tendency for bias in favour of older publications.^23^ To address this issue, we have also measured the number of citations per year (Table 1). It showed that the top 10 articles ranked by average citations per year were all published after 2000.

In conclusion, this study has provided a detailed analysis of top 100 most cited papers in head and neck radiology, providing insights into the most influential research in the field of head and neck radiology in the current time and allowing for the analysis and prediction of future trends. This bibliometric analysis also provides researchers with information on the characteristics of the highly cited papers in this field.
